# Art in Science Competition invites artworks to the annual exhibition on ISMB 2018 in Chicago

**DOI:** 10.12688/f1000research.14242.1

**Published:** 2018-03-19

**Authors:** Lonnie Welch, Bruno Gaeta, Diane E. Kovats, Milana Frenkel Morgenstern

**Affiliations:** 1 School of EECS, Ohio University, Athens, OH, USA; 2 School of Computer Science and Engineering, University of New South Wales, Sydney, New South Wales, Australia; 3 International Society for Computational Biology, Bathesda, MD, USA; 4 The Azrieli Faculty of Medicine, Bar-Ilan University , Safed, Israel

**Keywords:** art, science, competition

## Abstract

The International Society of Computational Biology and Bioinformatics (ISCB) brings together scientists from a wide range of disciplines, including biology, medicine, computer science, mathematics and statistics. Practitioners in these fields are constantly dealing with information in visual form: from microscope images and photographs of gels to scatter plots, network graphs and phylogenetic trees, structural formulae and protein models to flow diagrams, visual aids for problem-solving are omnipresent. The **ISCB Art in Science Competition** **2017 at the ISCB/ECCB 2017 conference in Prague** offered a way to show the beauty of science in art form. Past artworks in this annual exhibition at **ISMB** combined outstanding beauty and aesthetics with deep insight that perfectly validated the exhibit’s approach or went beyond the problem's solution. Others were surprising and inspiring through the transition from science to art, opening eyes and minds to reflect on the work being undertaken.

The International Society of Computational Biology and Bioinformatics (ISCB) brings together scientists from a wide range of disciplines, including biology, medicine, computer science, mathematics and statistics. Practitioners in these fields are constantly dealing with information in visual form: from microscope images and photographs of gels to scatter plots, network graphs and phylogenetic trees, structural formulae and protein models to flow diagrams, visual aids for problem-solving are omnipresent. The **ISCB Art in Science Competition 2017 at the ISCB/ECCB 2017 conference in Prague** offered a way to show the beauty of science in art form. Past artworks in this annual exhibition at **ISMB** combined outstanding beauty and aesthetics with deep insight that perfectly validated the exhibit’s approach or went beyond the problem's solution. Others were surprising and inspiring through the transition from science to art, opening eyes and minds to reflect on the work being undertaken.

Thirty unique pieces were showcased in the Art in Science competition at **ISCB/ECCB 2017 in Prague. There winners (reproduced below) were selected by the Art in Science review committee.**

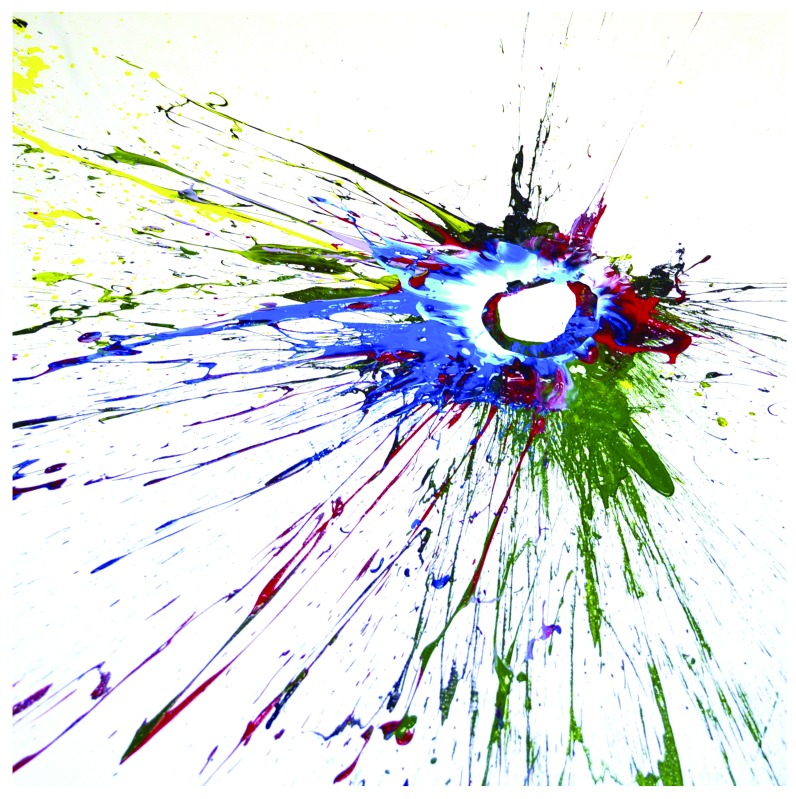



**Nick Schurch,** University of Dundee

**Chris Cole**, University of Dundee, United Kingdom

**Artwork title: ImpactFactor**

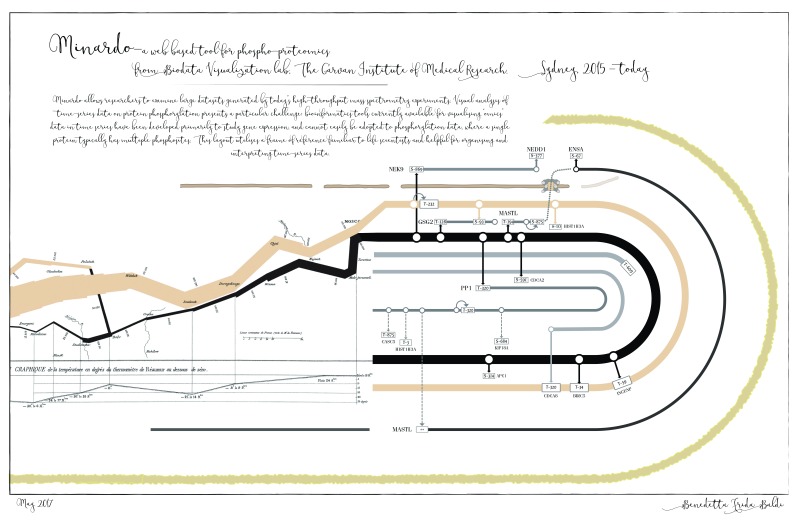



**Benedetta Frida Baldi,** The Garvan Institute of Medical Research

**Artwork title: Minard->o: how Napoleon marched into phosphoproteomic data**

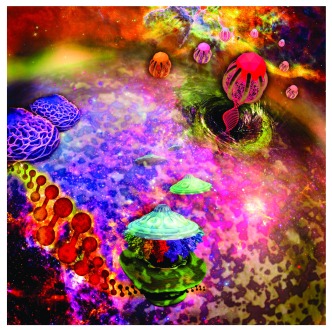



**Aviad Sivan,** Bar Ilan University

**Artwork title: Invasion by viruses**

ISCB invites submissions to the **2018 ISCB Art in Science competition**. All interested members may submit images that have been generated as part of a research project and other creative efforts that involve scientific concepts or employ scientific tools and methods. Submissions will be reviewed by a panel of judges. All submissions will be invited to **ISMB 2018** in Chicago Illinois, USA, to display their work. The winning submission will be announced during the ISCB Town Hall and presented with a $200 USD prize, as well as be the feature cover image for the *ISCB Fall Newsletter*.

**Submission page**: https://www.iscb.org/submissions/index.php?id=236

**The Art in Science review committee (2018):**

Milana Frenkel-Morgenstern, ***Bar-Ilan University*, chair**

Lonnie Welch, ***Ohio University*, co-chair**

Bruno Gaeta, ***UNSW Australia*, co-chair**

Mickey Kosloff, ***University of Haifa*, reviewer**

